# Arresting rampant dental caries with silver diamine fluoride in a young teenager suffering from chronic oral graft versus host disease post-bone marrow transplantation: a case report

**DOI:** 10.1186/1756-0500-7-3

**Published:** 2014-01-03

**Authors:** Chun-Hung Chu, Angeline Hui-Cheng Lee, Liwu Zheng, May Lei Mei, Godfrey Chi-Fung Chan

**Affiliations:** 1 Faculty of Dentistry, The University of Hong Kong, Hong Kong, SAR, China; 2 LKS Faculty of Medicine, The University of Hong Kong, Hong Kong, SAR, China

**Keywords:** Silver diamine fluoride, Rampant caries, Graft versus host disease

## Abstract

**Background:**

Rampant caries is an advanced and severe dental disease that affects multiple teeth. This case describes the management of rampant caries in a young teenager suffering from chronic oral graft versus host disease after allogeneic bone marrow transplantation.

**Case presentation:**

A 14-year-old Chinese boy suffering from β–thalassemia major was referred to the dental clinic for the management of rampant dental caries. An oral examination revealed pale conjunctiva, bruising of lips, and depapillation of tongue indicating an underlying condition of anemia. The poor oral condition due to topical and systemic immunosuppressants was seriously aggravated, and rampant caries developed rapidly, affecting all newly erupted, permanent teeth. The teeth were hypersensitive and halitosis was apparent. Strategies for oral health education and diet modification were given to the patient. Xylitol chewing gum was used to stimulate saliva flow to promote remineralization of teeth. Silver diamine fluoride was topically applied to arrest rampant caries and to relieve pain from hypersensitivity. Carious teeth with pulpal involvement were endodontically treated. Stainless steel crowns were provided on molars to restore chewing function, and polycarbonate crowns were placed on premolars, upper canines and incisors.

**Conclusion:**

This case report demonstrates success in treating a young teenager with severe rampant dental decay by contemporary caries control and preventive strategy.

## Background

Beta-thalassemias (β-thalassemias) are a group of genetic blood disorders caused by reduced or absent synthesis of the beta chains of hemoglobin, which are responsible for transportation of oxygen and carbon dioxide in the body. Although some patients can be clinically asymptomatic, there are patients who exhibit severe anemia. The total annual incidence of symptomatic individuals is estimated at 1 in 100,000 throughout the world [1]. Patients suffering from beta thalassemia often manifest severe anemia, poor growth, and skeletal abnormalities during infancy. Affected children will require regular, lifelong blood transfusions. Allogeneic hematopoietic cell (bone marrow) transplantation can be a curative treatment, but graft-versus-host disease (GvHD) can develop as a serious complication following bone marrow transplantation.

GVHD is not an uncommon complication following bone marrow transplant. In patients suffering from GVHD, their transplanted immune cells attack their body normal cells because the immune cells recognize their host as 'foreign’. GVHD can be manifested in acute or chronic from [2]. Acute GVHD is characterized by selective damage to the liver, skin, mucosa, and the gastrointestinal tract. Chronic GVHD also attacks the above organs, but it can also cause damage to other tissue and organs. Diagnosis and treatment of chronic graft-versus-host disease (GvHD) can be difficult and there are no UK guidelines on the diagnosis and management of chronic GvHD [2]. Chronic GvHD was traditionally defined as occurring more than 100 days after transplant [2]. The National Institutes of Health consensus conference proposed 2 subcategories for chronic GvHD, classic and overlap syndrome, based on clinical features rather than time of onset [2]. This proposal recognized that classical features of chronic GvHD could occur within 100 days of transplant and that features of acute and chronic GvHD could occur together.

Oral, chronic GvHD is very common and in some cases, the mouth may be the only area affected. Similar to chronic GvHD in other parts of the body, oral, chronic GvHD is variable and may range from not being painful at all to being so painful as to make it difficult to eat and speak. Because of the compromised medical condition, these children are at high risk of dental decay. The frequent intake of medication with syrup, and neglected oral hygiene, often allow development rampant caries within a short period of time.

Rampant caries refers to advanced and severe dental disease that affects multiple teeth [3], and is typically seen in children with neglected oral care, frequent sugar or syrup medicine intake, individuals with decreased salivary flow, and those with poor oral hygiene and drug addiction. Tooth surfaces that are ordinarily relatively caries-free are often affected. Despite the advances in dental treatment and prevention techniques, patients with rampant caries are very challenging to manage and the prognosis is not often satisfactory.

Fluoride agents such as sodium fluoride (NaF) or stannous fluoride (SnF) have been used for more than 50 years for caries prevention [4]. However, the effectiveness of NaF and SnF to arrest dental caries extending to dentine is not high. To arrest dental caries, 38% silver diamine fluoride (SDF) has been used in dentistry [5]. Thirty-eight percent SDF is a colorless solution that contains a high concentration of fluoride (44,800 ppm) [6]. Clinical studies reported that SDF is more effective than NaF to arrest dental caries [7,8]. A laboratory study also found that SDF has higher inhibitory effect on dentine demineralization than NaF [9]. It could be attributed to SDF’s ability to stimulate sclerotic dentine formation and germicidal effect on cariogenic bacteria by its silver salt [10].

SDF is topically applied on the cleaned and dried caries lesions by dental professionals using a microbrush. Removal of the soft carious before the SDF application is not necessary because such caries removal would not significantly affect the caries-arresting rate [8]. This treatment protocol is simple and is well accepted by young children [6]. As the treatment is noninvasive, the risk of spreading the infection is very low. Literature reviews concluded that silver diamine fluoride is simple, cost-effective, safe, and efficient with “equitable” agents for caries management [6,10]. Rosenblatt et al. [10] concluded that SDF is a caries control agent that can be used to help meet the World Health Organization Millennium Goals and fulfill the U.S. Institute of Medicine’s criteria for 21st century medical care. Milgrom and Chi [11] advocated that SDF therapy is an important prevention strategy. This paper describes the use of SDF to treat rampant caries in a young teenager suffering from major β-thalassemia and subsequently developing oral graft versus host disease after allogeneic bone marrow transplantation.

## Case presentation

**Figure 1:**
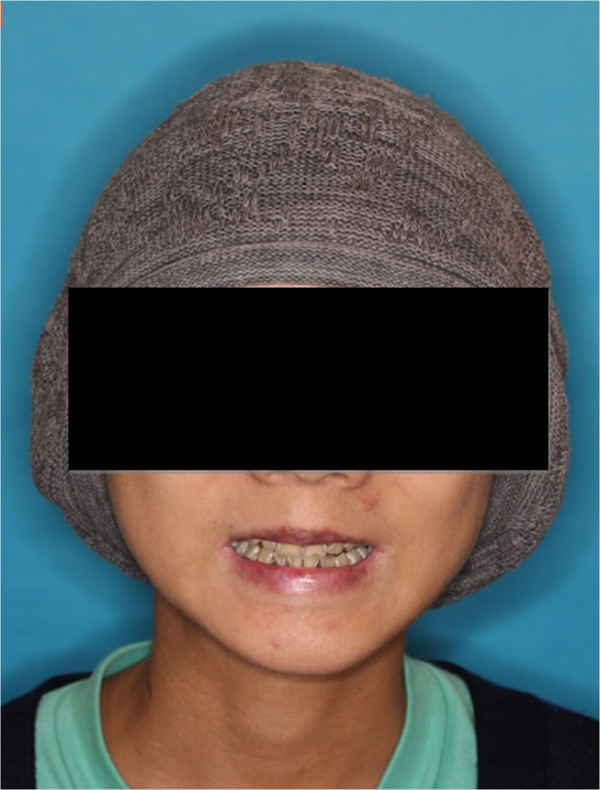
Full face photo.

A 14-year-old Chinese boy suffering from major β–thalassemia was referred to the dental clinic for treatment of rampant dental caries (Figure 1). He has had regular blood transfusions and iron chelation therapy since he was young. He underwent a matched, unrelated-donor bone-marrow transplant with successful engraftment, but developed moderate chronic oral graft versus host disease. His chief complaint was that he could not take cold drinks due to mucosal inflammation. However, he was not fully aware of his serious tooth-decay problem. His tooth brushing, if any, was very unsatisfactory because it was painful. His doctor prescribed chlorhexidine for mouth rinsing. However, he could not use it because of its burning sensation. Due to poor oral condition, the child was emaciated, weak, and fatigue. He did not have any regular dental care or visits for more than three years.

**Figure 2:**
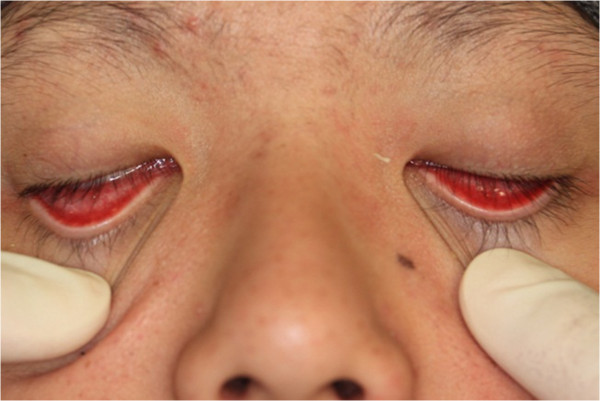
Pale conjunctiva.

**Figure 3:**
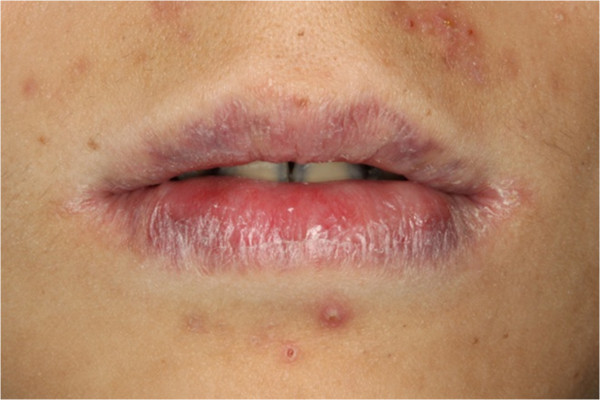
Bruising of the lips.

**Figure 4:**
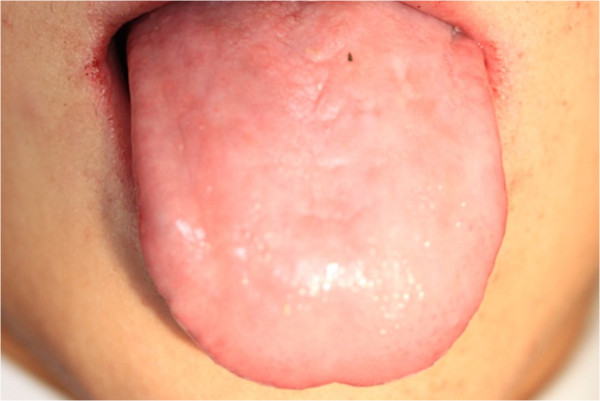
Depapillation of the tongue.

**Figure 5:**
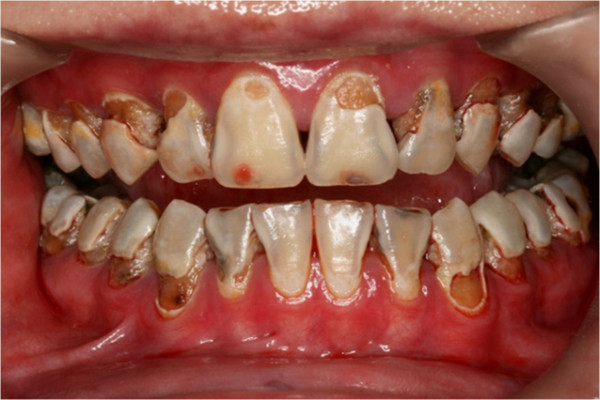
Pre-treatment intra-oral frontal view.

**Figure 6:**
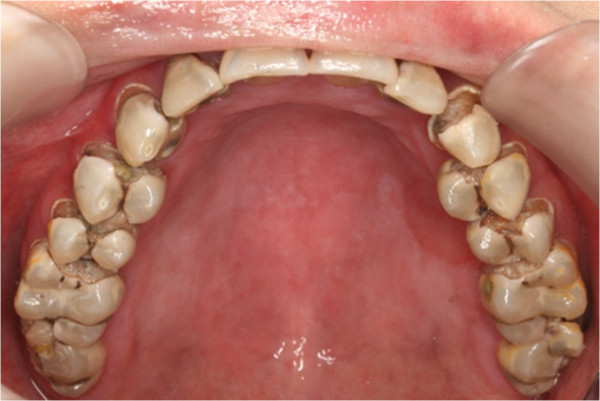
Pre-treatment upper occlusal view.

**Figure 7:**
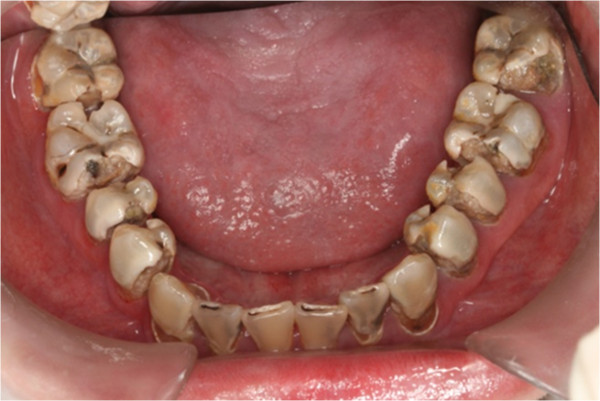
Pre-treatment lower occlusal view.

**Figure 8:**
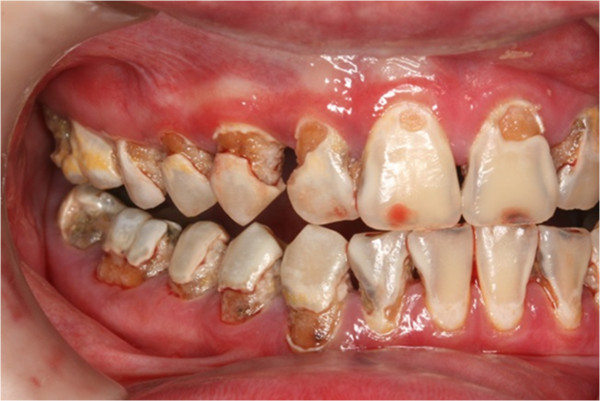
Pre-treatment left buccal view.

**Figure 9:**
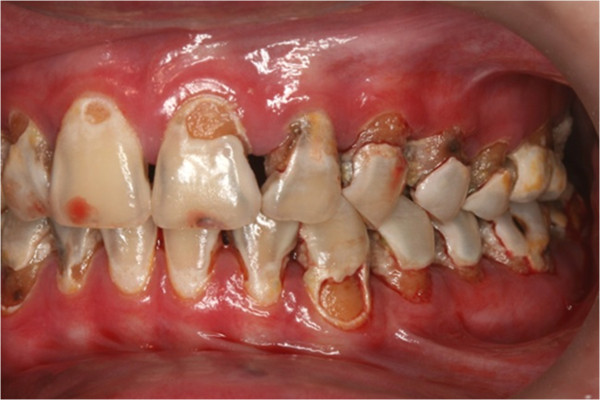
Pre-treatment right buccal view.

**Figure 10:**
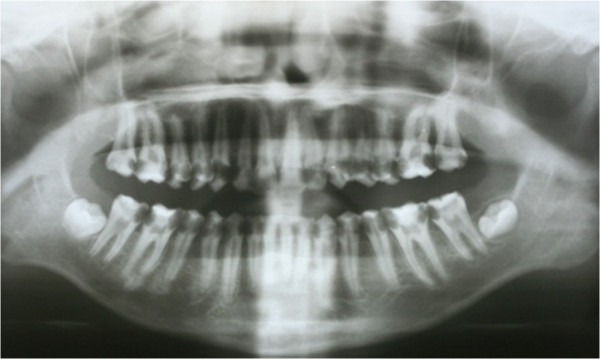
Pre-treatment panoramic radiograph.

**Figure 11:**
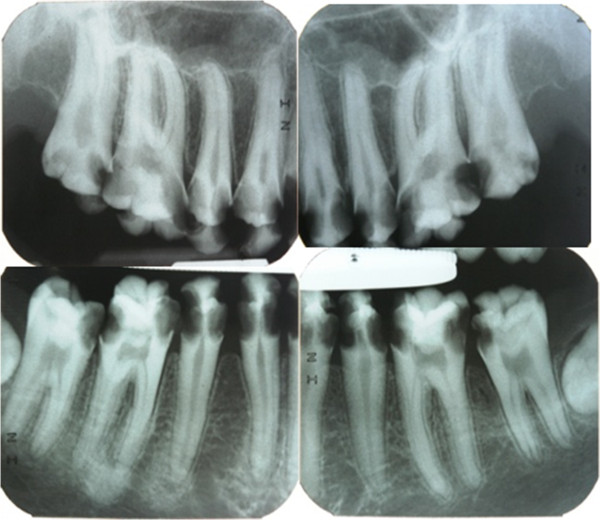
Pre-treatment periapical radiographs.

In clinical examination, he was lethargic and underweight. His conjunctiva was pale, a symptom of anemia (Figure 2). Oral examination revealed bruising of the lips (Figure 3) and depapillation of the tongue (Figure 4), indicating an underlying condition of anemia. The gingiva was markedly inflamed. No dental abscess was observed. The patient had a full, permanent dentition (except the third molar) with caries on all 28 erupted teeth (Figures 5, 6, 7, 8 and 9). The teeth were hypersensitive to cold-water rinsing (no air pressure) with a 3-in-1 syringe. The oral hygiene was extremely poor with thick plaque accumulation on the carious lesions. Halitosis was apparent. The mucosa was atrophic and thin, in particular on the buccal and labial surfaces. A panoramic radiograph (Figure 10) revealed extensive caries on the dentition. The dentine caries in most teeth were very soft. By electric pulp test, all maxillary premolars, anterior teeth, and lower-right premolars were found nonvital with apical radiolucencies on several nonvital premolars (Figure 11).

A sialometric assessment was performed on resting and stimulated salivary flow rate, and measurement of saliva acidity was done to assess the salivary function [12]. The patient was asked not to eat and drink within an hour before sialometric assessment. His unstimulated and stimulated saliva flow rates were 6.5 × 10^-4^ mL/s and 1.67 × 10^-3^ mL/s, respectively; and they were within the normal range. The salivary pH was found to be 6.5, which was lower than the normal value of 7.4.

The child was diagnosed with rampant caries and, remarkedly, with generalised gingivitis. The aim of the treatment plan was to promote his oral health, mastication, and aesthetics. The initial phase of the treatment was to relieve his discomfort, improve his oral hygiene, and arrest the progression of rampant caries. Definitive dental care should be considered when his medical condition becomes stable and his oral hygiene becomes satisfactory, when he reaches adulthood.

Tailored oral-health education was provided to the patient. To motivate the patient, the dentist decided to review his habits thoroughly, a diet analysis was performed, and he was advised to take less cariogenic nutritive foods such as rice porridge with minced pork. The patient found toothpaste difficult to use because of its texture and mint favor. He was therefore advised to brush his teeth at least twice daily by dipping an extra-soft toothbrush with 0.01 mm slim-tip bristles (Slim Soft, Colgate Sanxiao Co., Yangzhou, China) into 0.06% w/v chlorhexidine digluconate and sodium fluoride (250 ppm fluoride) (Corsodyl Daily Defence Mouthwash, GlaxoSmithKline, Middlesex, UK). An interdental brush was used to clean the interproximal area between adjacent teeth. Three professional applications of SDF (Saforide 38%, Toyo Seiyaku Kasei, Japan) were performed after plaque removal by gentle brushing by the dentist. The second application was provided two weeks afterward; and the third application was performed four weeks after the second application. Xylitol chewing gum was suggested at least six times daily to promote salivary flow.

**Figure 12:**
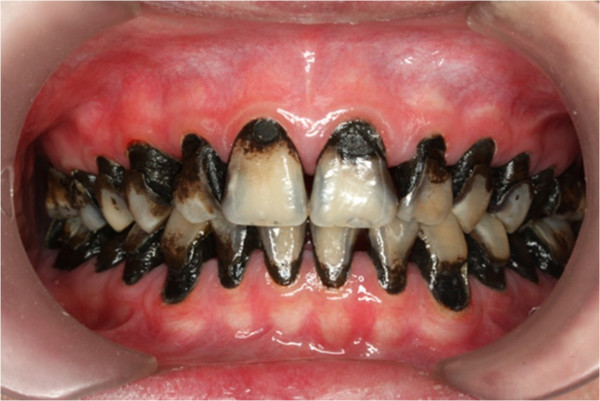
Front view after SDF treatment.

**Figure 13:**
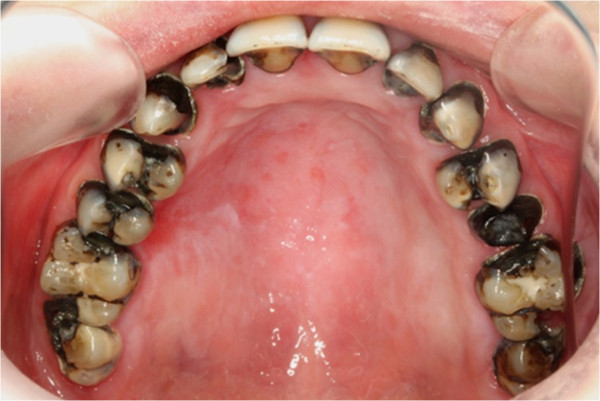
Upper occlusal view after SDF treatment.

**Figure 14:**
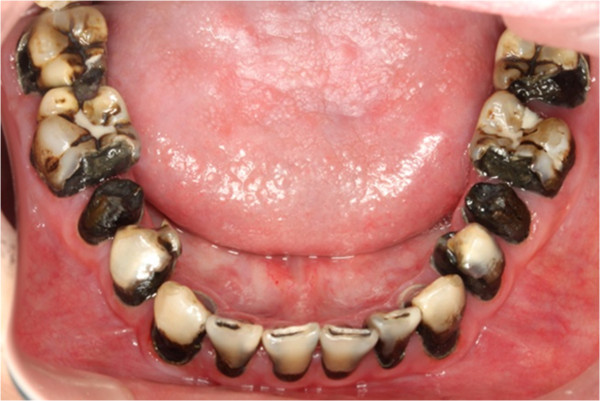
Lower occlusal view after SDF treatment.

**Figure 15:**
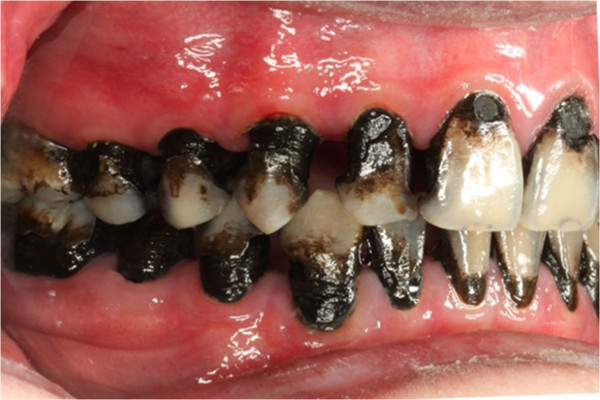
Left buccal view after SDF treatment.

**Figure 16:**
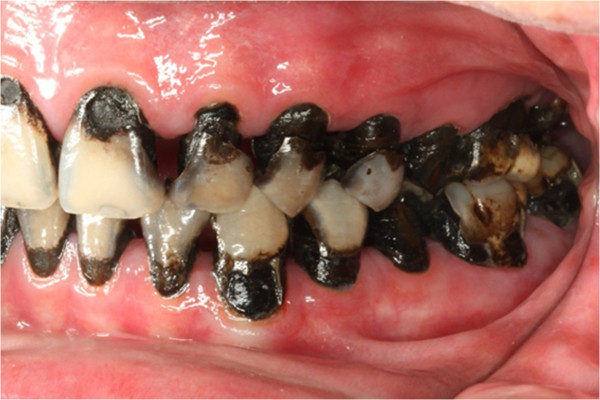
Right buccal view after SDF.

A follow-up visit was carried out four weeks afterward. The child reported no tooth hypersensitivity. Caries were found arrested and turned coal black in appearance (Figure 12). Some unsupported enamel chipped off from his teeth without any dental procedure. All of the enamel of the upper and lower-left second premolars, and two lower right premolars, was no longer existent, leaving a smooth, hardened, black dentine core behind (Figures 13, 14, 15 and 16). The oral hygiene of the patient was significantly improved and following gingival response was noticeable. He was advised to brush his teeth twice daily with fluoride toothpaste, and continue using a interdental toothbrush after every meal.

**Figure 17:**
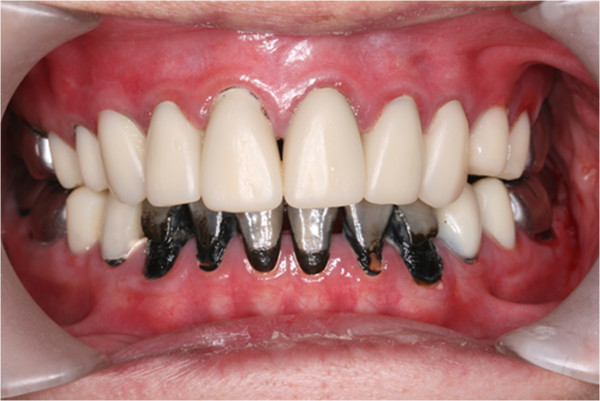
Frontal view after provisional crowns treatment.

**Figure 18:**
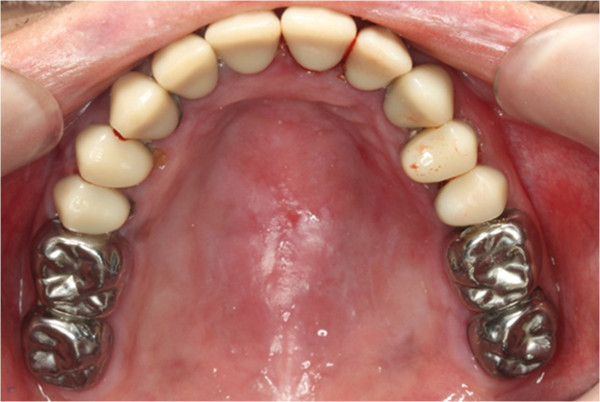
Upper occlusal view after provisional crowns treatment.

**Figure 19:**
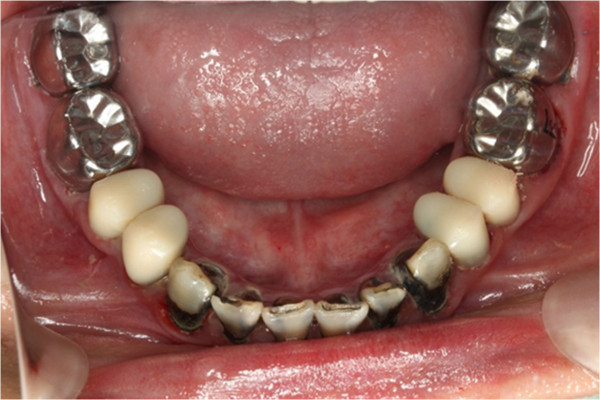
Lower occlusal view after provisional crowns treatment.

**Figure 20:**
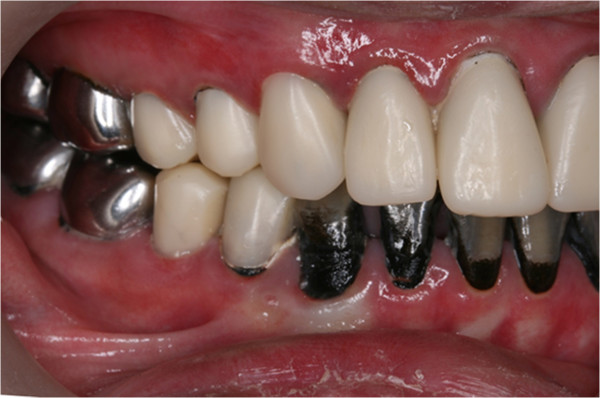
Left buccal view after provisional crowns treatment.

**Figure 21:**
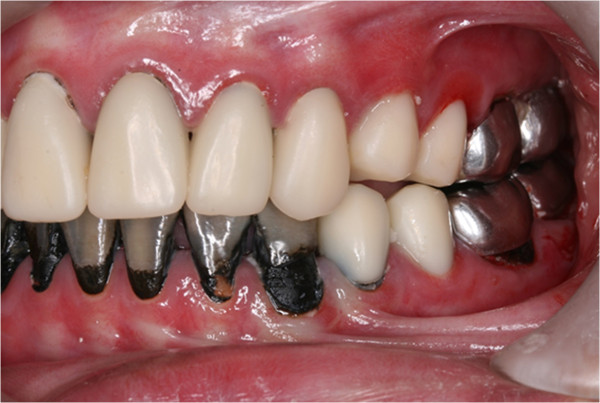
Right buccal view after provisional crowns treatment.

**Figure 22:**
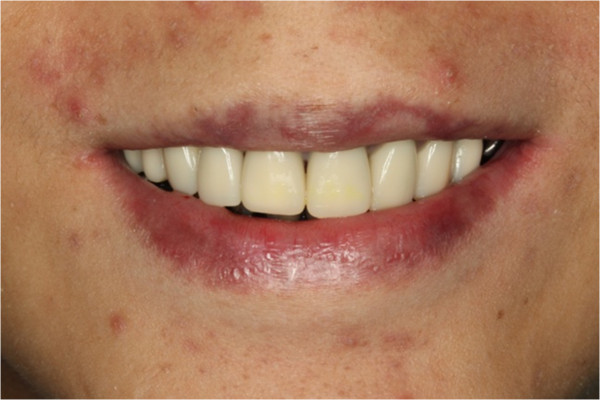
Smile photo after provisional crowns treatment.

After the caries became hardened, the first and second molars were stabilized with prefabricated posterior stainless steel crowns (Stainless Steel Permanent Molar Crown, 3 M ESPE Dental Products, St. Paul, MN U.S.A.) for mastication (Figures 17, 18, 19, 20 and 21). The upper anterior teeth and three of the premolars were restored with polycarbonate provisional crowns (Polycarbonate Prefabricated Crown, 3 M ESPE Dental Products, St. Paul, MN U.S.A.). Occlusal reduction was minimally performed for crown fitting. Crowns were cemented with restorative glass ionomer cement (Ketac Molar, 3 M ESPE Dental Products, St. Paul, MN U.S.A.). The mandibular anterior teeth were hard and smooth due to inadequate interdental space and they were left uncrowned at this stage. The discoloration of lower teeth was not shown when the boy smiled (Figure 22) and he was very satisfied with the provisional treatment and final result.

**Figure 23:**
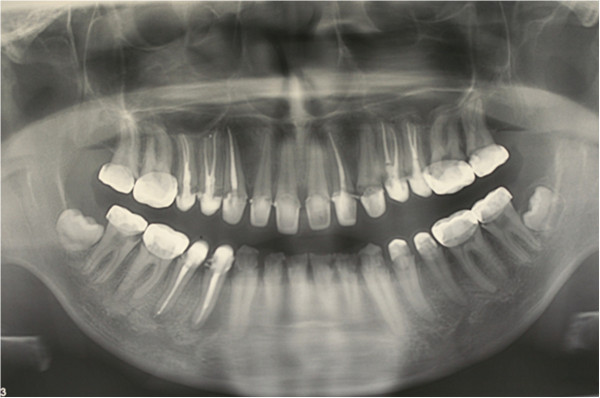
Post-endodontic treatment panoramic radiograph.

The upper right second premolars, the upper left canine, the two upper left premolars, and the lower right second premolars were nonresponsive to pulp vitality tests (EPT test). The patient was referred to an endodontist for root canal treatment on his nonvital teeth. The polycarbonate crowns were carefully removed and an access cavity was made through the glass ionomer (Ketac Molar) core. One-visit root canal therapy was performed and the crowns were recemented on the endodontically treated teeth (Figure 23).

## Discussion

β-thalassemias major is a severe hereditary disease that affects the production of hemoglobin in red blood cells. Patients with β-thalassemia major often suffer from severe anemia, skeletal abnormalities, and retarded growth and development. Regular, lifelong blood transfusions are necessary for the children with β-thalassemia major, and bone marrow transplants can be curative for some children. Chronic oral GvHD is one of the severe and major complications, post transplantation. They can be recalcitrant to convention topical and systemic immunosuppressive therapies. Due to the chronic oral GvHD, patients may have malnutrition due to alteration of taste sensation and tenderness to food and drink.

Due to the breakage of the oral mucosal barrier, and the use of potent systemic and topical immunosuppressants, opportunistic infection caused by bacteria, viruses, or fungus are common. This also aggravates the poor oral hygiene and dental caries development. The worst outcome is the development of oral cancer, which has been well documented. Since the original description by Izutse and associates [13], Sjogren’s like syndrome in patients with GVHD, presumably caused by salivary and ocular lymphocytic infiltrations, have been reported in many clinical studies [14]. This child patient presented severe dry eyes and seemingly dry mouth resulting in multiple dental and oral conditions. However, the resting and stimulated salivary flow rates were not significantly affected. He was encouraged to take chewing gum frequently to remineralize the carious lesions, lubricate the oral cavity, and minimize potential unfavorable taste alternations.

A comprehensive oral examination is critically important to patients with post transplantation because they are more susceptible to infections. Because of the immune-suppression and decreased inflammatory response, signs of infection may be muted and overlooked. Oral infections must be diagnosed and managed promptly because they may spread quickly, and systemic infections may manifest orally. Patients may develop symptoms of xerstomia, and the principle of management is to increase comfort and decrease caries risk. Cevimeline HCL, or cholinergic agonists pilocarpine, can be used to increase resting salivary flow rates; coating agents or artificial saliva can be used to moisten the mouth; sugarless candies and gums can be used to mechanically stimulate salivary flow; and topical fluoride can be used to minimize the risk of caries [14]. This child patient had a reasonable salivary flow rate and thus was encouraged to take chewing gum.

Despite the advances in clinical dentistry, rampant caries are still a challenging disease for a clinician to manage. This clinical case report demonstrated success at controlling rampant caries of a young teenager with SDF. Oral-health education was delivered through motivational interviewing, facilitating and engaging intrinsic motivation in order to change patient behavior [15]. Motivational interviewing is a patient-centered, purpose-oriented counseling, and it is more focused and goal-directed than nondirective counseling. By helping the child patient to explore and resolve ambivalence, and showing that the goal set is achievable, the child was motivated to change his behavior and improve his oral health.

Laboratory studies demonstrated that SDF can inhibit growth of cariogenic bacteria [16-18], promote remineralization [8], and increase the hardness of carious dentine [19]. Clinical studies found that SDF can prevent and arrest coronal and root caries [7,20]. SDF is also used to manage dentine hypersensitivity [21]. Rosenblatt and associates [10] performed a review on SDF and concluded that it is a safe, effective, efficient, and “equitable” caries-control agent that can be used to meet the World Health Organization Millennium Goals and fulfil the U.S. Institute of Medicine’s criteria for 21st century medical care. Milgrom and Chi 11] advocated that SDF therapy is an important prevention-centred caries management strategy during critical periods in early childhood. The use of silver diamine fluoride in clinical dentistry has been reported in countries in Asia, Australia, and South America such as Brazil, China, Cuba and Japan [6]. However, it is noteworthy that SDF is not yet cleared by the FDA for use in dentistry in the U.S. Some dentists in the U.S. suggest using 25% silver nitrate solution followed by 5% sodium fluoride varnish to arrest caries in children [22].

Advances in dental materials and technology have shortened the duration of endodontic treatment, such that the procedure, which conventionally requires two or more appointments, can now be performed in one visit. While multivisit endodontic treatment is considered the standard approach, there is no scientific evidence that it is better than a single-visit treatment. Completing endodontic treatment in a single appointment is known to be a convenience to patients and dentists, and avoids complications from leakage of the temporary seal that is needed between multiple appointments. Prashanth and associates [23] reported no significant difference in the success rate or postoperative pain, tenderness, and swelling when treated with either single-visit or multiple-visit endodontic therapy. Hence, one can readily integrate one-visit endodontic therapy into the routine clinical practice of dentistry.

Stainless steel crowns and polycarbonate crowns were used as provisional restorations to rehabilitate this patient’s mastication, speech, and aesthetics [24]. The treatment not only controlled caries but also enhanced patient satisfaction [25]. These restorations were found to have a profound effect on his self-esteem and confidence. Restorative glass ionomer, with superior mechanical properties, was used for cementation. Glass ionomer releases fluoride, which promotes remineralization of enamel and dentine [26].

## Conclusion

Rampant caries is a dental disease that is very challenging for dentists to manage. It can be seen in children with neglected oral care, or those suffering from severe systemic disease. The authors highlighted the use of silver diamine fluoride to control and arrest the rampant caries. Holistic and multidisciplinary treatments were also provided to improve the oral health and quality of life of the child patient.

## Consent

Written informed consent was obtained from the parents for publication of this case report and any accompanying images. A copy of the written consent is available for review by the series editor of this journal.

## Competing interests

The authors declare that they have no competing interests.

## Authors’ contributions

CHC delivered fluoride treatment and provisional crowns. AHCL provided root canal therapy. LWZ managed mucosal condition. MLM offered sialometric assessment and took clinical photographs. GCFC provided medical care. The 5 authors contributed equally on the manuscript. All authors read and approved the final manuscript.

## Authors’ information

Chun-Hung Chu is a general dentist who delivered fluoride treatment and provisional crowns. Angeline Hui-Cheng Lee is an endodontist who provided root-canal therapy. Liwu Zheng is a specialist in oral medicine who treated patient’s mucosal condition. May Lei Mei is a post-doctoral fellow who offered sialometric assessment and took clinical photographs. Godfrey Chi-Fung Chan is a pediatric hematology/oncology specialist who provided medical care. The authors contributed equally to the manuscript.
